# Arginine Availability in Reamed Intramedullary Aspirate as Predictor of Outcome in Nonunion Healing

**DOI:** 10.3390/biomedicines10102474

**Published:** 2022-10-03

**Authors:** Dennis M. Meesters, Karolina A. P. Wijnands, Hans M. H. van Eijk, Martijn Hofman, Frank Hildebrand, Jan P. A. M. Verbruggen, Peter R. G. Brink, Martijn Poeze

**Affiliations:** 1 Department of Surgery, Division of Trauma Surgery, Maastricht University Medical Center +, 6200 MD Maastricht, The Netherlands; 2 NUTRIM School for Nutrition and Translational Research in Metabolism, 6200 MD Maastricht, The Netherlands; 3 Department of Trauma and Reconstructive Surgery, University Hospital RWTH Aachen, 52074 Aachen, Germany

**Keywords:** nonunion, autologous bone grafting, nitric oxide, arginine, citrulline

## Abstract

Fracture healing and nonunion development are influenced by a range of biological factors. Adequate amino acid concentrations, especially arginine, are known to be important during normal bone healing. We hypothesize that bone arginine availability in autologous bone marrow grafting, when using the reamer-irrigator-aspirator (RIA) procedure, is a marker of bone healing capacity in patients treated for nonunion. Seventeen patients treated for atrophic long bone nonunion by autologous bone grafting by the RIA procedure were included and divided into two groups, successful treatment of nonunion and unsuccessful, and were compared with control patients after normal fracture healing. Reamed bone marrow aspirate from a site distant to the nonunion was obtained and the amino acids and enzymes relevant to arginine metabolism were measured. Arginine and ornithine concentrations were higher in patients with successful bone healing after RIA in comparison with unsuccessful healing. Ornithine concentrations and arginase-1 expression were lower in all nonunion patients compared to control patients, while citrulline concentrations were increased. Nitric oxide synthase 2 (*Nos2*) expression was significantly increased in all RIA-treated patients, and higher in patients with a successful outcome when compared with an unsuccessful outcome. The results indicate an influence of the arginine–nitric oxide metabolism in collected bone marrow, on the outcome of nonunion treatment, with indications for a prolonged inflammatory response in patients with unsuccessful bone grafting therapy. The determination of arginine concentrations and *Nos2* expression could be used as a predictor for the successful treatment of autologous bone grafting in nonunion treatment.

## 1. Introduction

Nonunion development occurs in 10–15% [1] of patients with long bone fractures. This incidence can increase up to 45% depending on risk factors such as fracture configuration and infection [2,3]. Next to a major decrease in quality of life, nonunion is accompanied by high socioeconomic costs caused by multiple surgical interventions needed for adequate treatment [4]. A regular treatment option for long bone nonunions is the use of autologous bone grafting by reamed-irrigation-aspiration (RIA) for the harvesting of bone and bone marrow, combined with the debridement of avital bone parts. This technique fulfills the requirements for adequate bone grafting stated in the Diamond Concept [5,6]. The general risk of persisting failure of bone healing after a bone grafting procedure for nonunion using the RIA procedure is around 10–18% [7,8,9]. Although the molecular pathogenesis of nonunion remains unclear, a better understanding may provide better approaches for its diagnosis and treatment.

It is known that disturbances in amino acid metabolism play a role in an inadequate bone healing response, especially amino acids relating to arginine–nitric oxide metabolism [10]. Nitric oxide (NO, solely produced during the conversion of arginine into citrulline by one of the nitric oxide synthases, NOSs) production is important during fracture healing because of its influence on the stimulation of bone cells to regulate bone remodeling [11], vascularization [12] and a possible stimulation of polyamine production as a precursor for collagen synthesis [13,14]. In vivo animal studies have already shown a localized [15] and temporal [16] expression of the different NOS isoforms in fracture tissue. Next to this, the absence of NOS isoforms results in diminished bone formation and nonunion development [17]. In humans, NOS isoforms were also expressed in mRNA (messenger ribonucleic acid) in callus samples [18], indicating an active role in the healing process.

We hypothesize that the arginine–NO metabolism in the human body plays a role in the molecular pathogenesis of abnormal bone healing and that measuring the concentrations of these amino acids and their related enzymes from the bone harvested during the RIA procedure is indicative of the success or failure of the nonunion treatment.

Hence, we investigate amino acid concentrations and relevant enzyme expressions in bone marrow obtained during the RIA procedure for autologous bone grafting in long bone nonunions, and compare them between successful outcomes and failures of this treatment. Patients with comparable fractures with normal fracture healing after fracture treatment were included as baseline control samples.

## 2. Materials and Methods

### 2.1. Patient Inclusion

This study was approved by the medical ethics committee of the Maastricht University Medical Center (permit METC04021). Written informed consent was obtained from all patients. Included, were patients which were admitted for surgery on atrophic long bone nonunions at the Department of Surgery in the Maastricht University Medical Center (MUMC+) and where the reamer-irrigator-aspirator procedure had been performed. The site for harvesting the autograft was always the femoral bone in these patients. When the nonunion was located in the femur, the ipsilateral femur was used for the harvesting of bone marrow. Atrophic nonunion was defined as a lack of radiological visible signs of healing of the fracture after 9 months from the initial trauma. seventeen patients were included and retrospectively divided into two groups. In the first group, nine patients achieved primary healing after the RIA procedure (bone healing within 6–9 months after surgery), and eight patients in the second group either had secondary success (one or more re-interventions after the primary RIA procedure to obtain healing of the nonunion) or a persisting absence of healing. As control tissue, trabecular bone samples were obtained during regular elective surgery in which there was access to the bone marrow in patients with healed femur or tibia fractures in order to remove osteosynthesis materials. Patient characteristics are presented in Table 1. The nonunion scoring system (NUSS) was used to classify the nonunion severity.

Bone marrow samples were collected directly after harvesting the marrow and snap frozen in liquid nitrogen in the operating theatre. Samples were stored at −80 °C until analysis.

### 2.2. High-Performance Liquid Chromatography Amino Acid Analysis

For the measurement of relevant amino acid concentrations, tissue samples were crushed in liquid nitrogen, deproteinized, homogenized and centrifuged as described before in detail [17]. The obtained supernatant was 100-fold diluted in water and 100 μL was placed in a WISP-style vial and placed in the chilled (4–8 °C) sample compartment of a Waters 717 plus autosampler (Waters Chromatography BV, Etten-Leur, The Netherlands). The amino acid analysis was performed after pre-column derivatization using *o*-phthaldialdehyde (Thermo Fischer Scientific), as described previously [19].

### 2.3. RNA Isolation and qPCR

Before RNA isolation, samples were crushed with a pestle and mortar in liquid nitrogen. To isolate total RNA, crushed samples were incubated, precipitated and centrifuged as described before. Pellets were then washed and dried before dissolving in diethylpyrocarbonate-treated water for subsequent cDNA synthesis.

For quantitative polymerase chain reaction (qPCR), iQ SYBR Green Supermix (Biorad Products, Hercules, CA, USA) and gene-specific forward and reverse primers were added to the cDNA. The cDNA was amplified using the MyIQ system (Biorad Products, Hercules, CA, USA) via a 3-step program: 40 cycles of denaturation (95 °C, 10 s), annealing (60 °C, 20 s) and elongation (70 °C, 20 s). Gene expression levels of *Nos2*, *Nos3,* and *Arg1* were determined using IQ5 software (Biorad Products, Hercules, CA, USA). The geometric mean of cyclophilin A (*Ppia*) and β-actin (*ActB*) expression levels was calculated and used as a normalization factor. All primers were acquired from Sigma-Aldrich (Zwijndrecht, The Netherlands). Primer sequences are shown in Table 2.

### 2.4. Statistical Analysis

Statistical analyses were performed using GraphPad Prism 6 (GraphPad, San Diego, CA, USA). Normality was checked using the Shapiro–Wilk test. All data are presented as means and standard error of the mean (SEM). Significance was calculated using one-way ANOVA testing with post hoc Bonferroni correction. *p*-values below 0.05 were considered as statistically significant. For regression analysis, SPSS 25.0 was used (IBM, Armonk, NY, USA). A multivariate procedure was used to provide a regression analysis of variance for dependent variable groups and with the factors BMI, age, NUSS and the arginine metabolism-related factors Nos- and Arginase 1-expression, and concentrations of arginine, citrulline and ornithine as covariates, presented as nonstandardized regression coefficients (B) (with SE).

## 3. Results

### 3.1. Patient Characteristics

The demographic characteristics of patients included in this study are presented in Table 1. A significant age difference was observed between patients with primary success and patients with refractory nonunion (*p* < 0.05). Although length and weight of the patients did not show significant differences, the body mass index (BMI) from patients in the control group was significantly lower in comparison with both the primary and secondary success after RIA treatment groups (both *p* < 0.05). The NUSS score was significantly higher in patients with primary success after treatment when compared with refractory nonunion patients. All other demographic factors (sex, smoking, alcohol and non-steroidal anti-inflammatory drug (NSAID) use, patient history of diabetes and the fracture location and Gustilo grade) did not show any significant differences between the groups.

### 3.2. Amino Acid Concentrations

**Figure 1:**
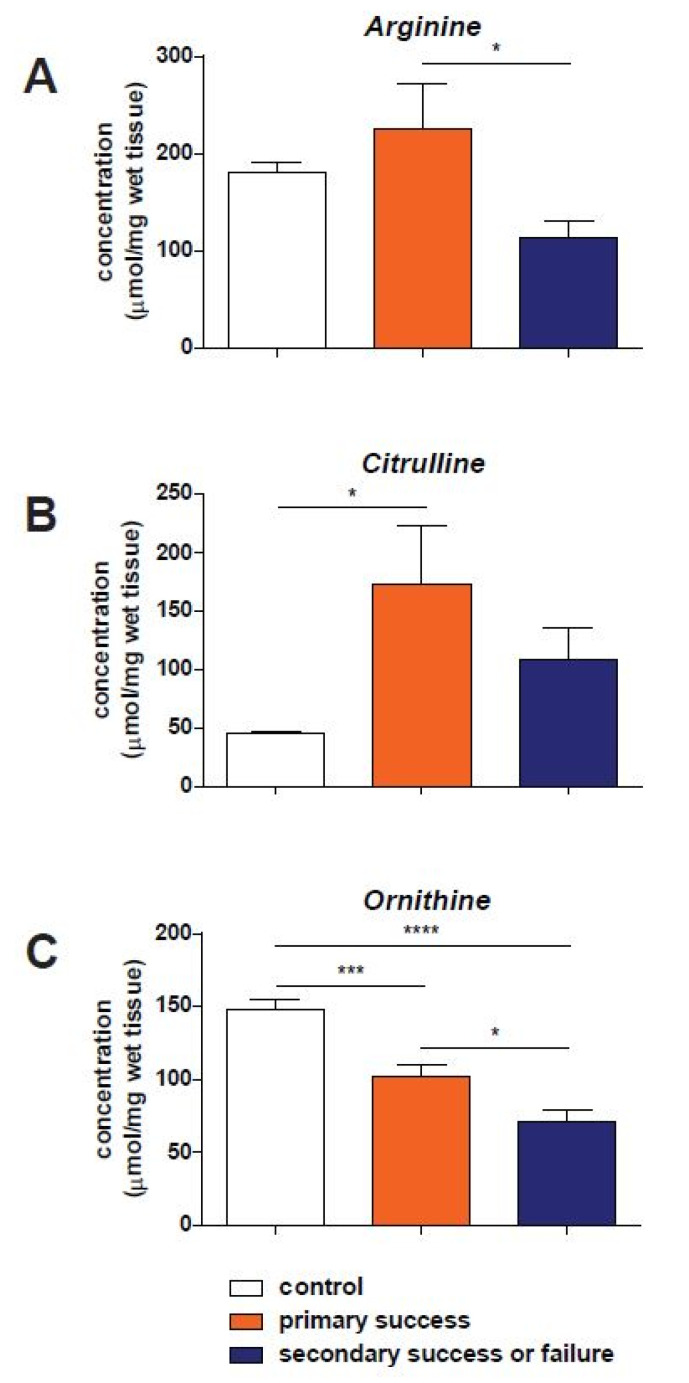
Concentrations of arginine (panel **A**), citrulline (**B**) and ornithine (**C**) in reamed intramedullary aspirate, presented as µmol/mg wet tissue. Results in control tissues are presented in the white bars. Samples obtained from patients with a primary successful RIA treatment are shown in orange and with an unsuccessful treatment in dark blue. *: *p* < 0.05; *** *p* < 0.001 and ****: *p* < 0.0001.

In Figure 1, concentrations of arginine, citrulline and ornithine measured in reamed intramedullary aspirate are shown. Arginine concentrations are significantly higher in patients in the primary success group compared to patients with a failure of the RIA treatment which needed one or more surgical re-interventions (*p* < 0.05, 225 ± 46.9 and 113 ± 17.2 µmol/mg wet tissue, respectively, Figure 1A). No significant differences were found when concentrations in both patient groups treated with RIA were compared with control samples.

Citrulline concentrations in samples of primary success patients (173 ± 50.3 µmol/mg wet tissue) and the secondary success and failure group (109 ± 26.7) were comparable. However, patients which achieved primary success after the RIA procedure showed significant higher citrulline levels compared to the control group (45.3 ± 3.49, *p* < 0.05, Figure 1B), while patients with a secondary success or failure had similar concentrations compared to control samples.

Ornithine concentrations also showed different concentrations between the three study groups (Figure 1C). Samples obtained from control patients showed significantly higher ornithine concentrations (148 ± 7.38 µmol/mg wet tissue) compared with primary success samples (102 ± 7.57, *p* < 0.001), as well as patients with failure of healing after RIA (71.5 ± 7.69, *p* < 0.0001). Further, patients with an initial success of the RIA procedure also showed higher ornithine levels compared to patients with failure of treatment or with the need for secondary surgical intervention (*p* < 0.05).

### 3.3. qPCR Analysis

**Figure 2:**
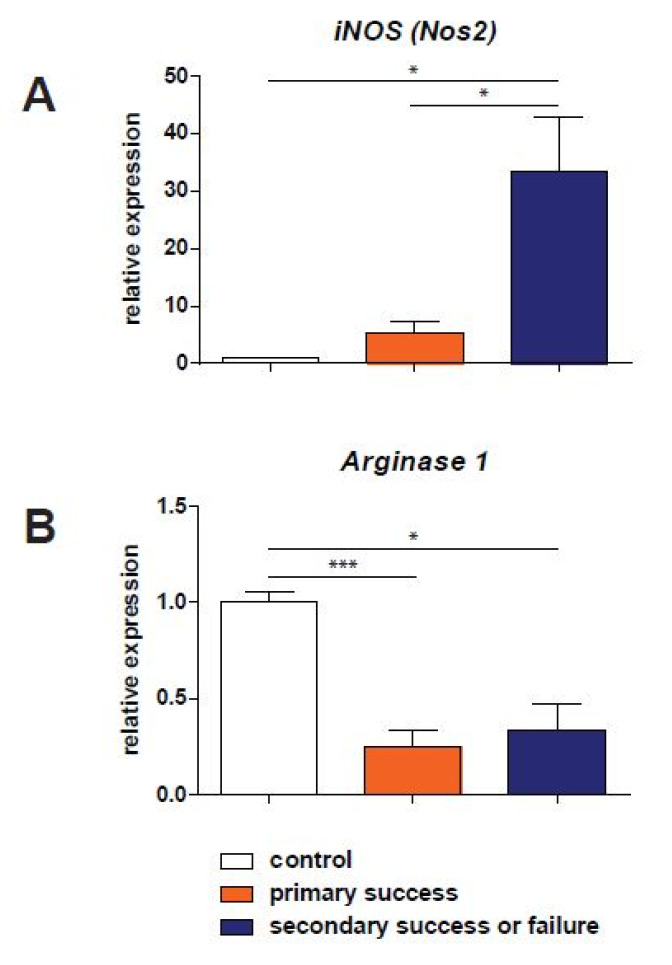
Relative expression of iNOS (Nos2, inducible nitric oxide synthase, panel (**A**)) and Arginase-1 (Arg1, panel (**B**)). Results in control tissue are represented in white bars. Samples obtained from patients with subsequently successful RIA treatment are shown in orange. The failure of the treatment group is presented in dark blue. Levels of significance: *: *p* < 0.05 and ***: *p* < 0.001).

The RNA expression of enzymes relevant to the arginine–NO metabolism is shown in Figure 2. Measurements of *Nos2* (inducible nitric oxide synthase) expression in trabecular bone from the reamed intramedullary aspirate showed a significant upregulation in patients with a successful RIA treatment as well as in patients where the procedure was not successful (both *p* < 0.05) when compared to samples obtained from control patients (Figure 2A). *Nos3*, the enzymatic isoform present in the endothelium of bone vasculature, was not detectable in all patients who were treated with the RIA procedure. In Figure 2B, the expression of *Arg1* is shown in the three study groups. A significant downregulation (0.24 and 0.34 of the values in control patients) is visible in both RIA-treated groups of patients when compared to control samples (*p* < 0.001 and *p* < 0.05 for primary success and the secondary success and failure group, respectively).

### 3.4. Regression Analysis

All variables were subsequently used as independent variables in a logistic regression analysis. The dependent variables of age, BMI, NUSS, arginine, citrulline and ornithine concentrations, and arginase-1 and iNOS expression were included as being significant predictors of outcome on univariate analysis. On multivariate analysis, iNOS was the only significant factor within these variables. Outlined below is the overview of the different significance levels and nonstandardized regression coefficients (with SE): age (B 0.001, SE 0.215, p 0.514), BMI (B −0.014, SE 0.006, p 0.057), NUSS (B 0.003, SE 0.002, p 0.202), arginine concentration (B −0.001, SE 0.000, p 0.179), citrulline concentration (B 0.000, SE 0.000, p 0.357), ornithine concentration (B −0.003, SE 0.002, p 0.146), arginase-1 expression (B 0.421, SE 0.190, p 0.057) and iNOS expression (B 0.025, SE 0.002, *p* < 0.001).

## 4. Discussion

This is the first study in which biomarkers of the arginine–nitric oxide metabolism have been clinically evaluated in trabecular bone harvested during RIA from patients treated for nonunion of the long bones. Concentrations of both the amino acids arginine and ornithine were higher in samples obtained from patients that had a successful bone healing after bone grafting when compared with those that had an unsuccessful healing. Relevant enzymes, *Nos2* and *Arg1*, showed differences in samples obtained from the reamed intramedullary aspirate when compared to bone marrow obtained from patients with an initial normal fracture healing, while Nos2 could be differentiated between successful and primarily failed nonunion treatment. This predictive value might lead towards its possible future use as a biomarker in predicting nonunion healing outcome.

Generally, 10–15% of all long bone fractures fail to heal adequately [1,20,21], resulting in the development of nonunions with major functional impairment and a decrease in the quality of life for these patients, which is accompanied by a high socioeconomic burden [2,4]. A wide range of possible biomarkers that can be used as predictors for the development of nonunions have been investigated [22,23], however, our current study shows, for the first time, the use of possible biomarkers as a predictor for a successful nonunion treatment.

One of the major components for the treatment of long bone nonunion is autologous bone grafting. Bone grafting is used with proven effectiveness; the transplantation of sufficient cells, scaffold and growth factors from other, non-affected locations to the non-consolidating bone can stimulate new bone healing. While the effectiveness of the autologous bone grafting in promoting consolidation of nonunion is high, it can vary considerably among patients, from 80 to 90% [21,24]. Knowledge in predicting factors is limited, but encompass both clinical and biological markers. A number of studies reported either specific (e.g., scaphoid or tibia) or general (e.g., NUSS score) clinical and radiological factors for predicting outcome after the treatment of nonunions [25,26,27]. In addition, Granchi et al. [28] showed a decrease in the biochemical bone turnover markers, bone-specific alkaline phosphatase and C-terminal propeptide, of type I procollagen, which were observed during treatment failure. The current study adds to the evidence that biomarkers, in addition to clinical markers, can have prognostic value in the treatment of patients with a nonunion.

Differences in molecular patterns in bone grafts between patients with success and failure at a site distant from the nonunion may indicate that systemic molecular pathologies are partly responsible for the failure of nonunion treatment and that nonunion is not a purely local metabolic problem. The decreased concentrations of arginine in the nonunion callus tissue in a previous study [10] and in the harvested bone in the patients with a failed response to the bone harvesting treatment seem to be an indication for this hypothesis.

Sufficient formation of nitric oxide, a free radical, influences vascular reactivity [12] and stimulates bone cells to regulate bone remodeling during fracture repair [11]. Through the subsequent formation of ornithine, it also stimulates the production of polyamines, which are precursors for collagen synthesis [13]. Previous studies have already shown that callus tissue and plasma samples of patients with nonunions have abnormally low concentrations of the amino acids arginine, citrulline and ornithine when compared to normal healed and acute fractures [10], with corroborating data also found in animal experiments [17,29,30]. The importance of the NOS isoforms during fracture healing has, up until now, mainly been investigated in in vivo models of fracture repair [15,16,17,18]. Callus tissue from femoral fractures in rats showed a different temporal and spatial expression of these isoforms during the healing process. The inducible NOS (*Nos2*) is present during the first inflammatory reaction after sustaining the fracture, and is localized along the edge of the periosteal callus [15,18]. *Nos3*, which is constitutively expressed, is mainly present during later phases of fracture healing in cells lining the blood vessels [15,18]. The fact that *Nos1* is expressed during the remodeling stages [16] led us to not focus on this enzyme during the present study. The correlation of NOSs and fracture repair is further emphasized by experiments in which non-selective NOS inhibitors are supplemented to animals after inducing a fracture which lead to a decrease in the cross-sectional callus area [18]. Furthermore, genetic deletion of *Nos2* or *Nos3* leads to a decreased bone formation and subsequent nonunion formation in mice [17,31].

In the current study, we found a significantly increased *Nos2* expression in bone marrow aspirate obtained during the RIA procedure in patients where the bone grafting procedure had an unsuccessful outcome, compared to patients with adequate bone healing after the RIA procedure. An increased Nos2 expression suggests a prolonged inflammatory response (i.e., stimulation of NF-κΒ), resulting in the production of proinflammatory cytokines as interleukin-1, tumor necrosis factor-α (TNF-α) and TNF-β. Since a disturbed chronic inflammatory response during fracture healing might result in a delayed union or nonunion formation, this could be the reason that the clinical response to the RIA treatment is inadequate [32,33]. The significantly lower arginine concentrations which coincide with the higher *Nos2* expression may indicate depletion of this amino acid by an increased catabolic response of the patient [34].

Arginase-1 is the enzyme converting arginine into ornithine and subsequently leading to collagen synthesis via the production of polyamines. RIA procedures resulting in a successful bone healing, as well as in unsuccessful healing, showed a 3- to 4-fold lower expression of arginase-1 compared to normally healed fractures. This might reflect the anabolic response of the bone during the healing process which was initially the cause of the nonunion development and the subsequent need for surgical intervention in the fracture healing process. Additionally, the importance of arginase-1 is also reflected by the lower ornithine concentrations measured in the reamed intramedullary aspirate in the patients with nonunions (Figure 2).

A number of factors are known predictors of nonunion development. While the NUSS score is a known factor in patients with a fracture to define the risk of subsequently developing a nonunion [35], the study found the NUSS score to also be a predictor of the success rate for the treatment of the nonunion. Interestingly, compared to the NUSS score, the activity of the inflammatory response in the grafted material obtained by RIA was an even better predictor of therapy success.

A limitation of this study is the heterogeneity of different characteristics in patients included in the current investigation. In particular, defect size, NUSS [26,36], Gustilo classification and fracture localization show a wide range for a relatively low number of patients. Ideally, a large cohort of patients with similar characteristics in all groups is needed to minimalize the possiblity of confounding effects, and this study should therefore be regarded a hypothesis-generating pilot study to determine whether the underlying heterogeneity in our population influenced the amino acid concentrations. With regards to the amino acid concentrations, and especially arginine, there are several conditions that can alter the concentrations of arginine and related amino acids in plasma, such as diabetes mellitus, inflammation and renal or hepatosplanchnic dysfunction, due to the compromising function on the availability and conversion of citrulline into arginine [37,38,39,40]. In addition, other host factors, such as smoking or alcohol use and the use of NSAIDs, could possibly influence fracture healing negatively [2]. As shown in Table 1, these factors did not significantly differ between the control group and both nonunion groups, also supporting the clinical relevance of the presented results.

## 5. Conclusions

In conclusion, the results presented in the current study indicate: (1) an influence of the arginine–nitric oxide metabolism in bone grafts harvested by reamed intramedullary aspirate on the successful outcome of the autologous bone (marrow) grafting as treatment for long bone nonunions; (2) additionally, the concentration of the relevant amino acids and enzymes linked to this metabolic pathway might be used as biomarkers which could be advantageous to the prediction of outcome in addition to the clinical parameters which are already available.

## Figures and Tables

**Table 1:** Patient characteristics.

	Normal Bone Healing (Control Group) N = 8	Nonunion with Primary Success N = 9	Refractory Nonunion N = 8	Significance
Age (years)	58 (30–66)	64 (51–86)	44 (18–71)	*p* = 0.03 *
Male/Female	4/4	3/6	5/3	N.S.
Length (cm)	174 (166–191)	172 (160–187)	176 (165–192)	N.S.
Weight (kg)	80 (65–94)	83 (60–108)	81 (62–108)	N.S.
BMI (kg/cm^2^)	20.0 (19.1–30.4)	28.0 (22.9–31.2)	26.5 (19.0–39.7)	*p* < 0.05 ^
Alcohol use (yes/no)	3/5	5/4	1/7	N.S.
Smoking (yes/no)	6/2	5/4	6/2	N.S.
NSAID use (yes/no)	2/6	1/8	2/6	N.S.
DM (yes/no)	2/6	2/7	3/5	N.S.
Localization (n) Femur Tibia Humerus Radius	4 4 0 0	2 3 3 1	5 1 2 0	n.a.
Defect size (mm)	n.a.	30.4 (6–84)	50.4 (8–155)	N.S.
Gustilo (n) 0 1 2 3	8 0 0 0	5 2 0 2	2 1 1 4	
NUSS score (0–100)	n.a.	64 (51–86)	44 (18–71)	*p* = 0.03 *
Time between fracture and sampling (days)	2 (1–4)	499 (65–1143)	655 (191–2331)	

Abbreviations: BMI: body mass index; NSAID: non-steroidal anti-inflammatory drugs; NUSS: nonunion scoring scale; DM: diabetes mellitus; N.S.: not significant; n.a.: not applicable; *: significance between primary success and refractory nonunion; ^: significance of both groups compared to control.

**Table 2:** Primer sequences for quantitative polymerase chain reaction.

Gene	Name	Sequence (5′ → 3′)
*Ppia*	Cyclophylin-A (Fw) Cyclophylin-A (Rev)	CTCGAATAAGTTTGACTTGTGTTT CTAGGCATGGGAGGGAACA
*ActB*	Beta-actin (Fw) Beta-actin (Rev)	GCTGTGCTACGTCGCCCTG GGAGGAGCTGGAAGCAGCC
*Nos2*	iNOS (Fw) iNOS (Rev)	TTGCAAGCTGATGGTCAAGATC CAACCCGAGCTCCTGGAA
*Nos3*	eNOS (Fw) eNOS (Rev)	TTAATGTGGCCGTGTTGCA CTCTTGATGGAAGACAGGAGTTAGG
*Arg1*	Arginase-1 (Fw) Arginase-1 (Rev)	CGCCAAGTCCAGAACCATAGG TCTCAATACTGTAGGGCCTTCTT

Abbreviations: Fw: forward; Rev: reverse.

## Data Availability

The data presented in this study are available on request from the corresponding author. The data are not publicly available due to privacy and ethical reasons.

## References

[1] Mills L.A., Aitken S.A., Simpson A. (2017). The risk of non-union per fracture: Current myths and revised figures from a population of over 4 million adults. Acta Orthop..

[2] Bishop J.A., Palanca A.A., Bellino M.J., Lowenberg D.W. (2012). Assessment of compromised fracture healing. J. Am. Acad. Orthop. Surg..

[3] Karladani A.H., Granhed H., Karrholm J., Styf J. (2001). The influence of fracture etiology and type on fracture healing: A review of 104 consecutive tibial shaft fractures. Arch. Orthop. Trauma Surg..

[4] Kanakaris N.K., Giannoudis P.V. (2007). The health economics of the treatment of long-bone non-unions. Injury.

[5] Giannoudis P.V., Einhorn T.A., Marsh D. (2007). Fracture healing: The diamond concept. Injury.

[6] Masquelet A.C., Begue T. (2010). The concept of induced membrane for reconstruction of long bone defects. Orthop. Clin. N. Am..

[7] Dawson J., Kiner D., Gardner W., Swafford R., Nowotarski P.J. (2014). The reamer-irrigator-aspirator as a device for harvesting bone graft compared with iliac crest bone graft: Union rates and complications. J. Orthop. Trauma.

[8] Belthur M.V., Conway J.D., Jindal G., Ranade A., Herzenberg J.E. (2008). Bone graft harvest using a new intramedullary system. Clin. Orthop. Relat. Res..

[9] Stafford P.R., Norris B.L. (2010). Reamer-irrigator-aspirator bone graft and bi Masquelet technique for segmental bone defect nonunions: A review of 25 cases. Injury.

[10] Wijnands K.A., Brink P.R., Weijers P.H., Dejong C.H., Poeze M. (2012). Impaired fracture healing associated with amino acid disturbances. Am. J. Clin. Nutr..

[11] Chae H.J., Park R.K., Chung H.T., Kang J.S., Kim M.S., Choi D.Y., Bang B.G., Kim H.R. (1997). Nitric oxide is a regulator of bone remodelling. J. Pharm. Pharmacol..

[12] Corbett S.A., McCarthy I.D., Batten J., Hukkanen M., Polak J.M., Hughes S.P. (1999). Nitric oxide mediated vasoreactivity during fracture repair. Clin. Orthop. Relat. Res..

[13] Vittur F., Lunazzi G., Moro L., Stagni N., de Bernard B., Moretti M., Stanta G., Bacciottini F., Orlandini G., Reali N. (1986). A possible role for polyamines in cartilage in the mechanism of calcification. Biochim. Biophys. Acta.

[14] Xia W., Szomor Z., Wang Y., Murrell G.A. (2006). Nitric oxide enhances collagen synthesis in cultured human tendon cells. J. Orthop. Res. Off. Publ. Orthop. Res. Soc..

[15] Zhu W., Murrell G.A., Lin J., Gardiner E.M., Diwan A.D. (2002). Localization of nitric oxide synthases during fracture healing. J. Bone Miner. Res..

[16] Zhu W., Diwan A.D., Lin J.H., Murrell G.A. (2001). Nitric oxide synthase isoforms during fracture healing. J. Bone Miner. Res..

[17] Meesters D.M., Neubert S., Wijnands K.A., Heyer F.L., Zeiter S., Ito K., Brink P.R., Poeze M. (2016). Deficiency of inducible and endothelial nitric oxide synthase results in diminished bone formation and delayed union and nonunion development. Bone.

[18] Diwan A.D., Wang M.X., Jang D., Zhu W., Murrell G.A. (2000). Nitric oxide modulates fracture healing. J. Bone Miner. Res..

[19] van Eijk H.M., Rooyakkers D.R., Deutz N.E. (1993). Rapid routine determination of amino acids in plasma by high-performance liquid chromatography with a 2–3 microns Spherisorb ODS II column. J. Chromatogr..

[20] Mills L.A., Simpson A.H. (2013). The relative incidence of fracture non-union in the Scottish population (5.17 million): A 5-year epidemiological study. BMJ Open.

[21] Giannoudis P.V., Gudipati S., Harwood P., Kanakaris N.K. (2015). Long bone non-unions treated with the diamond concept: A case series of 64 patients. Injury.

[22] Pountos I., Georgouli T., Pneumaticos S., Giannoudis P.V. (2013). Fracture non-union: Can biomarkers predict outcome?. Injury.

[23] Wildemann B., Ignatius A., Leung F., Taitsman L.A., Smith R.M., Pesantez R., Stoddart M.J., Richards R.G., Jupiter J.B. (2021). Non-union bone fractures. Nat. Rev. Dis. Primers.

[24] Giannoudis P.V., Einhorn T.A., Schmidmaier G., Marsh D. (2008). The diamond concept--open questions. Injury.

[25] Ramamurthy C., Cutler L., Nuttall D., Simison A.J., Trail I.A., Stanley J.K. (2007). The factors affecting outcome after non-vascular bone grafting and internal fixation for nonunion of the scaphoid. J. Bone Joint Surg. Br..

[26] Calori G.M., Phillips M., Jeetle S., Tagliabue L., Giannoudis P.V. (2008). Classification of non-union: Need for a new scoring system?. Injury.

[27] Zura R., Della Rocca G.J., Mehta S., Harrison A., Brodie C., Jones J., Steen R.G. (2015). Treatment of chronic (>1 year) fracture nonunion: Heal rate in a cohort of 767 patients treated with low-intensity pulsed ultrasound (LIPUS). Injury.

[28] Granchi D., Gomez-Barrena E., Rojewski M., Rosset P., Layrolle P., Spazzoli B., Donati D.M., Ciapetti G. (2017). Changes of Bone Turnover Markers in Long Bone Nonunions Treated with a Regenerative Approach. Stem Cells Int..

[29] Meesters D.M., Hannemann P.F., van Eijk H.M., Schriebl V.T., Brink P.R., Poeze M., Wijnands K.A. (2020). Enhancement of fracture healing after citrulline supplementation in mice. Eur. Cell Mater..

[30] Kdolsky R.K., Mohr W., Savidis-Dacho H., Beer R., Puig S., Reihsner R., Tangl S., Donath K. (2005). The influence of oral L-arginine on fracture healing: An animal study. Wien. Klin. Wochenschr..

[31] Baldik Y., Diwan A.D., Appleyard R.C., Fang Z.M., Wang Y., Murrell G.A. (2005). Deletion of iNOS gene impairs mouse fracture healing. Bone.

[32] Claes L., Recknagel S., Ignatius A. (2012). Fracture healing under healthy and inflammatory conditions. Nat. Rev. Rheumatol..

[33] Schindeler A., McDonald M.M., Bokko P., Little D.G. (2008). Bone remodeling during fracture repair: The cellular picture. Semin. Cell Dev. Biol..

[34] Wijnands K.A., Hoeksema M.A., Meesters D.M., van den Akker N.M., Molin D.G., Briede J.J., Ghosh M., Kohler S.E., van Zandvoort M.A., de Winther M.P. (2014). Arginase-1 deficiency regulates arginine concentrations and NOS_2_-mediated NO production during endotoxemia. PLoS ONE.

[35] van Basten Batenburg M., Houben I.B., Blokhuis T.J. (2019). The Non-Union Scoring System: An interobserver reliability study. Eur. J. Trauma Emerg. Surg..

[36] Calori G.M., Colombo M., Mazza E.L., Mazzola S., Malagoli E., Marelli N., Corradi A. (2014). Validation of the Non-Union Scoring System in 300 long bone non-unions. Injury.

[37] Flynn N.E., Meininger C.J., Haynes T.E., Wu G. (2002). The metabolic basis of arginine nutrition and pharmacotherapy. Biomed. Pharmacother..

[38] Windmueller H.G., Spaeth A.E. (1981). Source and fate of circulating citrulline. Am. J. Physiol..

[39] Wu G., Morris S.M. (1998). Arginine metabolism: Nitric oxide and beyond. Biochem. J..

[40] Moinard C., Cynober L. (2007). Citrulline: A new player in the control of nitrogen homeostasis. J. Nutr..

